# Dietary practices and supplement use among CrossFit® participants

**DOI:** 10.1080/15502783.2022.2086016

**Published:** 2022-07-04

**Authors:** Matthew Brisebois, Samuel Kramer, Keston G. Lindsay, Chien-Ting Wu, James Kamla

**Affiliations:** a Department of Human Performance and Health, The University of South Carolina Upstate, Spartanburg, SC, USA; b International Vitamin Corporation, Greenville, SC, USA; c University of Colorado Colorado Springs, Department of Human Physiology and Nutrition, Colorado Springs, CO, USA

**Keywords:** high-intensity functional training, HIFT, questionnaire, survey, nutrition

## Abstract

**Background:**

CrossFit® is a popular high-intensity functional training program. CrossFit® participants may practice popular diets or consume dietary and sports supplements to support their health or physical pursuits, but the specific dietary and supplement practices of CrossFit® participants remain unknown.

**Methods:**

An electronic questionnaire was developed to collect data on practice of popular diets (i.e. Paleo and The Zone Diet®), dietary and sports supplement use, reasons for practicing a diet or using supplements, sources of information on diets and supplements, and various beliefs associated with nutrition among CrossFit® participants.

**Results:**

Of the 2,576 complete responses (female 51.9%, male 48.1%, age 39.4 ± 11.1 years, body mass index 26.1 ± 3.9 kg/m^2^), 695 (27%) reported being a CrossFit® trainer or coach and 1,392 (54%) reported competing, or planning to compete, in CrossFit® or other fitness competitions. The average years of CrossFit® experience were 5.3 ± 3.1 years, and the average frequency of CrossFit® participation was 4.5 ± 1.1 days/week. Most participants (60.1%) reported practicing a particular diet. Macro Counting (18.6%), Intermittent Fasting (7.7%), and Paleo (6.1%) were the most frequently reported diets. The top reasons for practicing a diet were to improve overall health (45.6%), decrease body fat (29.2%), and improve CrossFit® performance (25.2%). The top sources of dietary information were the Internet (47.5%), coach/trainer (28.7%), and nutritionist/dietitian (26.2%). Most participants (67.3%) reported “Urine Color” as the best method to assess hydration. Additionally, most participants (82.2%) consumed at least one supplement, with protein (51.2%), creatine (22.9%), and pre-workout/energy (20.7%) being most popular. The top reasons for consuming supplements were to improve recovery (52.6%), improve overall health (51.4%), and increase muscle mass/strength (41.7%). The top sources of information on supplements were the Internet (53.1%), coach/trainer (27.0%), and peer-reviewed research (23.0%).

**Conclusions:**

A large proportion of CrossFit® participants may practice popular diets or consume supplements with the intention of improving health or performance. These findings may support future research on the effects of various dietary patterns and supplements on CrossFit® performance.

## 1. Introduction

CrossFit® is one of the most popular workout programs in the world, with over 11,000 registered gyms worldwide [1]. CrossFit® is a high-intensity functional training (HIFT) program that incorporates a variety of coordinated multi-joint movements performed at high relative intensities [2,3]. Many individuals perform CrossFit® for the health benefits, social interaction, and physical challenges it presents [4]. In addition to serving as a workout program, some individuals pursue CrossFit® as a competitive sport. Over 290,000 individuals participated in the 2022 CrossFit® Open [5], an online competition where CrossFit® participants around the world submit their scores in a series of workouts. CrossFit® also hosts a Level 1 Certificate Course, which includes instruction on CrossFit®’s training method, exercises, and nutrition, which is a prerequisite for becoming a CrossFit® coach [6].

In addition to implementing a workout program, CrossFit® provides dietary recommendations for their community via their website [7] and Level 1 Certificate Course [6]. CrossFit® summarizes a healthy eating pattern as consuming “ … meat and vegetables, nuts and seeds, some fruit, little starch, and no sugar,” a recommendation similar to the Paleolithic (Paleo) diet [7,8]. Furthermore, participants should “keep intake to levels that will support exercise but not body fat” [7]. CrossFit® also recommends The Zone Diet® (40% carbohydrate, 30% protein, and 30% fat) as a starting point to determine the individual’s optimal macronutrient distribution for physical performance [9,10]. However, each CrossFit® gym operates as its own autonomous entity, and the leaders of CrossFit® do not require them to teach a specific dietary pattern. Maxwell et al. [11] reported that CrossFit® coaches commonly recommend Paleo and The Zone Diet® for their clients; however, scientific data on the diets practiced among members of the CrossFit® community are lacking. CrossFit® has also recommended thirst as the best measure for assessing hydration status [12,13], but it is unknown whether CrossFit® participants follow this guidance.

CrossFit® participants may also use dietary or sports supplements to improve their health or performance. Dietary and sports supplements may be defined as products consumed with the purpose of improving health and/or performance that are not intended to treat, prevent, or cure disease, such as vitamins, creatine, or protein powder [14]. The global dietary supplement market continues to grow and is projected to reach $298.5 billion by 2027 [15]. It has been reported that recreationally active adults and athletes may use supplements to improve their health, body composition, or workout performance [16,17]. Anecdotally, CrossFit® participants are likely to use supplements, but there are currently scarce scientific data to support these claims.

Many individuals around the world perform CrossFit®, but there are little scientific data on the specific diets they practice or supplements they use. The purpose of this study was to characterize the dietary and supplement practices of CrossFit® participants. The findings may help direct discussion on dietary and supplement recommendations for members of the CrossFit® community and guide future research on how specific diets and supplements impact CrossFit® performance.

## 2. Materials and methods

### 2.1 Questionnaire

The questionnaire used in this study was adapted from previous validated questionnaires on supplement use in athletes [18–20] and was reviewed for content and clarity by a Registered Dietitian, a Certified Strength and Conditioning Specialist, three CrossFit® coaches, and the owner of a retail nutrition store.

Data were collected anonymously via electronic software (Qualtrics®, Provo, UT). The questionnaire contained 23 items that queried the participants on demographic and anthropometric information, CrossFit® participation habits, coaching status, whether they participate in competitions, the diet they primarily practiced over the past six months, and the name and brand of supplements they used at least twice per week over the past six months. CrossFit® affiliate gym names were not included in the survey. Participants were asked to select their primary reasons for practicing a diet and using supplements and their primary sources of information on diets and supplements (participants were allowed to select multiple responses to these questions). Participants were also asked several affective questions, including which nutrient they believe is most important for CrossFit® performance, how important they believe nutrition and supplements are for CrossFit® performance, and which method they believe is best for assessing hydration: changes in body weight, thirst, urine color, or unsure. Due to the timing of data collection, participants were asked to report if their exercise, diet, or supplement habits had been affected by the COVID-19 pandemic.

Before reporting on supplement use, participants were provided the following information: “Dietary and sports supplements are products consumed in the form of tablets, capsules, softgels, gelcaps, liquids, powders, or gummies with the purpose of improving health and/or performance. Examples of dietary and sports supplements include vitamins, minerals, amino acids, creatine, protein powders, fish/omega-3 oils, fat burners, pre-workouts, probiotics, mixtures of natural ingredients to support health (adrenal, brain, immune system, joint, or sleep support), or other natural substances (CBD oil, turmeric, melatonin, glucosamine, beetroot juice, tart cherry, green tea extract, etc.)” [14,20].

The questionnaire was pilot tested with male (*n* = 9) and female (*n* = 6) volunteers from various CrossFit® gyms who were asked not to participate in the final questionnaire. Participants took the questionnaire twice, two weeks apart. The average time to complete the questionnaire was 5.8 ± 1.6 min. The data were coded and cleaned before measuring test–retest reliability. All but three items had at least moderate agreement (Cohen’s kappa >0.41 for categorical variables) or strong correlation (Pearson *r* >0.8 for continuous variables). The pilot study participants were interviewed on these items, and alterations were made to enhance clarity. The final questionnaire may be viewed in Additional File 1.

The study methods were approved by the Institutional Review Board of the University of South Carolina. The first page of the questionnaire was an Informed Consent, which was completed by all participants. The questionnaire was advertised using a flyer that was distributed to owners of CrossFit® gyms electronically or in-person. The questionnaire was also advertised by a news outlet that publishes CrossFit®-related articles [21]. Data collection took place May–July 2021. Due to the wide reach of the advertising methods, it was not feasible to determine a response rate.

The questionnaire link was opened 3424 times and 2924 individuals started the questionnaire. Of those individuals, 188 (6.4%) did not complete the questionnaire and 160 (5.5%) did not provide adequate details about the supplements they used. Therefore, 2576 (88.1%) complete responses were recorded and analyzed.

### 2.2 Statistical analysis

Statistical analysis was performed using IBM SPSS Statistics, version 28 (SPSS Inc., Chicago, IL, USA). Multiple correspondence analysis (MCA) to map the relationships between diets and supplements was performed using R version 4.0.4 [22]. The *FactoMineR* package [23] was used for analysis and the *factoextra* package [24] for visualization. Body mass index (BMI) was calculated as weight (kg)/height (m)^2^. The data were checked for outliers and a normal distribution before analysis. Descriptive statistics were used to report frequencies and percentages. Associations between demographic variables and practicing a diet or using supplements were analyzed with chi-square tests. Continuous variables, such as age or years of experience, were categorized into groups for chi-square tests. Continuous variables were compared using independent *t*-tests. Statistical significance was set at *P* < .05.

## 3. Results

### 3.1 Sample characterization

Of the 2576 participants, 1238 (48.1%) were male and 1338 (51.9%) were female. The mean years of CrossFit® experience were 5.26 ± 3.07 years, and the mean frequency of CrossFit® participation was 4.51 ± 1.11 days/week. The mean age was 39.36 ± 11.07 years, and the mean BMI was 26.14 ± 3.92 kg/m^2^. Males had a higher average BMI (27.03 ± 3.39 vs. 25.30 ± 4.19 kg/m^2^, *P* < .001) and more years of CrossFit® experience (5.41 ± 3.15 vs. 5.13 ± 2.99 years, *P* = .022) than females. Six-hundred ninety-five (27%) participants reported being a CrossFit® trainer or coach. CrossFit® coaches had lower average BMI (25.77 ± 3.30 vs. 26.27 ± 4.12 kg/m^2^, *P* = .004), more years of CrossFit® experience (6.98 ± 2.83 vs. 4.63 ± 2.91 years, *P* < .001), and performed CrossFit® more days per week (4.83 ± 0.96 vs. 4.39 ± 1.13 days, *P* < .001) than non-coaches. Additionally, 1392 (54%) participants reported that they compete or were planning to compete in CrossFit® or other fitness competitions. Those who reported competing or planning to compete were younger (37.86 ± 11.29 vs. 41.10 ± 10.55 years, *P* < .001), had lower BMI (25.98 ± 3.66 vs. 26.32 ± 4.20 kg/m^2^, *P* = .027), more years of CrossFit® experience (5.41 ± 3.01 vs. 5.09 ± 3.13 years, *P* = .010), and performed CrossFit® more days per week (4.78 ± 1.01 vs. 4.19 ± 1.13 days, *P* < .001) than those who did not. When asked about their reasons for performing CrossFit®, 2459 (95.5%) reported “Health,” 2324 (90.2%) reported “Fun/Enjoyment,” 2310 (89.7%) reported “Improve/Maintain Physical Appearance,” and 2106 (78.3%) reported “Social Interaction/Community.” When asked if the COVID-19 pandemic had affected their frequency of CrossFit® participation over the past six months, 585 (22.7%) reported “Yes.”

Most participants performed CrossFit® in the United States (*n* = 2304, 89.4%). There was at least one participant from each of the 50 states and Washington, D.C., with the highest proportion of participants from Texas (*n* = 177, 6.9%), California (*n* = 164, 6.4%), and Florida (*n* = 131, 5.1%). Two hundred seventy-two (10.6%) participants performed CrossFit® outside the United States, with the greatest representation from Canada (*n* = 64, 2.5%), the United Kingdom (*n* = 35, 1.4%), and Australia (*n* = 26, 1.0%).

### 3.2 Associations between sample characteristics and practicing a diet or using supplements

**Figure 1.**
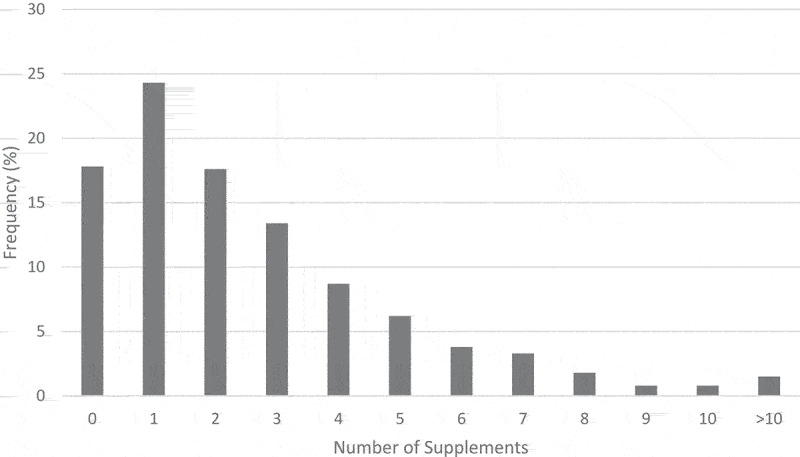
Number of supplements used at least twice per week by the participants over the past six months.

Characteristics of the participants, along with frequencies who reported practicing a diet or using supplements and their relationships between sample characteristics, may be viewed in Table 1. Most participants reported practicing a specific diet (*n* = 1547, 60.0%) or using at least one supplement twice per week (*n* = 2118, 82.2%) over the past six months. Participants used an average of 2.62 ± 2.64 supplements (Figure 1). The participant’s sex, age, BMI, days of CrossFit® per week, coaching status, and participation in competitions were all significantly associated with practicing a specific diet or using at least one supplement. Females were more likely to practice a specific diet (*P* < .001) and less likely to use supplements (*P* = .034) compared to males, although the number of supplements used between sexes was not significantly different. Participants who practiced a diet were older than those who did not (39.7 ± 10.6 vs. 38.8 ± 11.8 years, *P* = .026). Participants who exercised more days per week were more likely to practice a diet (4.6 ± 1.1 vs. 4.4 ± 1.1 days/week, *P* < .001) and use supplements (4.6 ± 1.1 vs. 4.3 ± 1.1 days/week, *P* < .001). Participants who practiced a diet also used more supplements than those who did not (2.86 ± 2.71 vs. 2.26 ± 2.49, *P* = .002). Participants who reported being a CrossFit® coach were more likely to practice a diet (*P* = .004), use supplements (*P* = .003), and use more supplements (2.97 ± 2.85 vs. 2.49 ± 2.55, *P* = .039) compared to non-coaches. Participants who reported competing, or planning to compete, in competitions were more likely to practice a diet (*P* < .001), use supplements (*P* < .001), and use more supplements (2.94 ± 2.74 vs. 2.24 ± 2.47, *P* < .001) than those who did not. Years of CrossFit® experience was also significantly associated with supplement use (*P* = .02), with users having more years of experience than non-users (5.4 ± 3.1 vs. 4.9 ± 3.1 years, *P* = .002). Three-hundred thirty-two (12.9%) participants reported having their dietary practices affected by the COVID-19 pandemic, which was more common among those who practiced a diet (*P* < .001). One-hundred seventy-two (6.7%) participants reported having their supplement use affected by the COVID-19 pandemic, which was more common among those who reported using supplements (*P* < .001).

**Table 1.** Characteristics of the participants and relationship between practicing a diet or using supplements.

	Total % (*n*)	Practices a diet			Uses supplements		
		Yes % (*n*)	No % (*n*)	*P*	Yes % (*n*)	No % (*n*)	*P*
Total	100 (2576)	60.1 (1547)	39.9 (1029)		82.2 (2117)	17.8 (459)	
Sex							
Male	48.1 (1238)	55.2 (683)	44.8 (555)	<.001	83.8 (1038)	16.2 (200)	.034
Female	51.9 (1338)	64.6 (864)	35.4 (474)		80.6 (1079)	19.4 (259)	
Age							
15–19 yrs	1.1 (29)	45.2 (14)	54.8 (17)	<.001	71.0 (22)	29.0 (9)	.021
20–29 yrs	17.6 (454)	53.0 (241)	47.0 (214)		80.4 (366)	19.6 (89)	
30–39 yrs	36.6 (943)	60.2 (568)	39.8 (375)		84.5 (797)	15.5 (146)	
40–49 yrs	25.8 (665)	65.4 (435)	34.6 (230)		82.1 (546)	17.9 (119)	
50–59 yrs	13.4 (344)	64.2 (221)	35.8 (123)		82.8 (285)	17.2 (59)	
60–69 yrs	4.7 (120)	49.2 (59)	50.8 (61)		73.3 (88)	26.7 (32)	
70+ yrs	0.7 (18)	50.0 (9)	50.0 (9)		72.2 (13)	27.8 (5)	
Body mass index^1^							
Underweight	0.3 (9)	77.8 (7)	22.2 (2)	ns	55.6 (5)	44.4 (4)	.011
Normal	40.6 (1047)	60.4 (632)	39.6 (415)		83.6 (875)	16.4 (172)	
Overweight	46.1 (1188)	61.0 (725)	39.0 (464)		82.4 (980)	17.6 (209)	
Obese	12.3 (316)	55.7 (176)	44.3 (140)		77.2 (244)	22.8 (72)	
Years of CrossFit®							
0–2 yrs	19.5 (503)	56.7 (285)	43.3 (218)	ns	78.1 (393)	21.9 (110)	.020
3–5 yrs	38.6 (994)	59.6 (592)	40.4 (402)		82.0 (815)	18.0 (179)	
6–8 yrs	25.9 (666)	61.4 (409)	38.6 (257)		85.0 (566)	15.0 (100)	
9–11 yrs	12.6 (325)	63.4 (206)	36.6 (119)		81.8 (266)	18.2 (59)	
12+ yrs	3.3 (86)	63.2 (55)	36.8 (32)		88.5 (77)	11.5 (10)	
CrossFit® frequency							
1–2 d/wk	4.1 (105)	50.5 (53)	49.5 (52)	<.001	81.9 (86)	18.1 (19)	<.001
3–4 d/wk	38.2 (985)	56.6 (558)	43.4 (427)		77.6 (764)	22.4 (221)	
5+ d/wk	57.1 (1470)	63.3 (933)	36.7 (540)		85.3 (1256)	14.7 (217)	
CrossFit® coach							
Yes	27.0 (695)	64.6 (449)	35.4 (246)	.004	85.9 (597)	14.1 (98)	.003
No	73.0 (1881)	58.4 (1098)	41.6 (783)		80.8 (1520)	19.2 (361)	
Planning to compete							
Yes	54.0 (1392)	63.2 (880)	36.8 (512)	<.001	86.5 (1204)	13.5 (188)	<.001
No	46.0 (1184)	56.3 (667)	43.7 (517)		77.1 (913)	22.9 (271)	
Pandemic affecteddietary practices							
Yes	12.9 (332)	72.3 (240)	27.7 (92)	<.001	-	-	-
No	87.1 (2244)	58.2 (1307)	41.8 (937)		-	-	
Pandemic affectedsupplement use							
Yes	6.7 (172)	-	-	-	92.4 (159)	7.6 (13)	<.001
No	93.3 (2404)	-	-		81.6 (1956)	18.4 (442)	
Uses supplements							
Yes	82.2 (2117)	62.2 (1317)	37.8 (800)	<.001	-	-	-
No	17.8 (459)	50.1 (230)	49.9 (229)		-	-	
Practices a diet							
Yes	60.1 (1547)	-	-	-	85.1 (1317)	14.9 (230)	<.001
No	39.9 (1029)	-	-		77.7 (800)	22.3 (229)	

### 3.3 Dietary practices and associated factors

The diets reported by the participants may be viewed in Table 2. The most frequently reported diets were Macro Counting (*n* = 479, 18.6%), Intermittent Fasting (*n*= 198, 7.7%), and Paleo (*n* = 156, 6.1%). Three of the diets presented as options (DASH Diet, Dukan Diet, and South Beach Diet) had no responses. Over 40 different diets were described in the “Other” category. Some of the most prevalent responses were as follows: Eating healthy, clean, whole, or unprocessed foods (*n* = 30), eating a combination of diets (i.e. Macro Counting plus Paleo, *n* = 22), 800 g Challenge (*n* = 14), low carbohydrate (*n* = 12), a diet prescribed by a dietitian or nutritionist (*n* = 10), Working Against Gravity (*n* = 8), and the CrossFit® recommendation (“Eat meat and vegetables, nuts and seeds, some fruit, little starch, and no sugar,” *n* = 4).

**Table 2.** Diets practiced by the participants and relationships between sex, coaching status, and supplement use.

Diet	Total % (*n*)	Sex			CrossFit® Coach			Uses supplements		
		Male % (*n*)	Female % (*n*)	*P*	Yes % (*n*)	No % (*n*)	*P*	Yes % (*n*)	No % (*n*)	*P*
Macro Counting	18.6 (479)	14.4 (178)	22.5 (301)	<.001	21.6 (150)	17.5 (329)	.018	20.8 (440)	8.5 (39)	<.001
IF	7.7 (198)	11.1 (137)	4.6 (61)	<.001	7.5 (52)	7.8 (146)	ns	7.6 (161)	8.1 (37)	ns
Paleo	6.1 (156)	6.3 (78)	5.8 (78)	ns	7.9 (55)	5.4 (101)	.016	6.0 (127)	6.3 (29)	ns
RP	5.0 (129)	4.2 (52)	5.8 (77)	ns	5.2 (36)	4.9 (93)	ns	5.6 (119)	2.2 (10)	.002
Gluten-Free	3.1 (80)	0.8 (10)	5.2 (70)	<.001	3.0 (21)	3.1 (59)	ns	3.0 (64)	3.5 (16)	ns
Ketogenic Diet	2.1 (54)	2.4 (30)	1.8 (24)	ns	2.2 (15)	2.1 (39)	ns	2.0 (43)	2.4 (11)	ns
Mediterranean	1.5 (39)	1.7 (21)	1.3 (18)	ns	1.7 (12)	1.4 (27)	ns	1.3 (28)	2.4 (11)	ns
The Zone Diet®	1.5 (38)	1.6 (20)	1.3 (18)	ns	3.0 (21)	0.9 (17)	<.001	1.4 (30)	1.7 (8)	ns
Vegan	1.5 (38)	1.4 (17)	1.6 (21)	ns	1.4 (10)	1.5 (28)	ns	1.6 (33)	1.1 (5)	ns
Vegetarian	1.5 (39)	1.3 (16)	1.7 (23)	ns	0.7 (5)	1.8 (34)	.045	1.3 (28)	2.4 (11)	ns
Whole 9	1.2 (30)	1.1 (13)	1.3 (17)	ns	1.0 (7)	1.2 (23)	ns	1.3 (27)	0.7 (3)	ns
Flexitarian	1.0 (25)	0.8 (10)	1.1 (15)	ns	0.7 (5)	1.1 (20)	ns	1.0 (21)	0.9 (4)	ns
Pescatarian	0.9 (22)	0.6 (7)	1.1 (15)	ns	0.4 (3)	1.0 (19)	ns	1.1 (5)	0.8 (17)	ns
Carnivore	0.7 (18)	1.1 (14)	0.3 (4)	.011	0.6 (4)	0.7 (14)	ns	0.6 (13)	1.1 (5)	ns
WW	0.7 (17)	0.2 (3)	1.0 (14)	.012	0.0 (0)	0.9 (17)	.012	0.6 (13)	0.9 (4)	.041
Atkins Diet®	0.2 (6)	0.3 (4)	0.1 (2)	ns	0.1 (1)	0.3 (5)	ns	0.7 (3)	0.1 (3)	ns
Other	6.9 (179)	5.9 (73)	7.9 (106)	ns	7.5 (52)	6.8 (127)	ns	7.1 (50)	6.3 (29)	ns

Females were more likely than males to practice Macro Counting (*P* < .001), Gluten-Free (*P* < .001), and Weight Watchers® (*P* = .012) diets, and males were more likely to practice Intermittent Fasting (*P* < .001) and Carnivore (*P* = .011) diets. CrossFit® coaches were more likely to practice Macro Counting (*P* = .018), Paleo (*P* = .016), and The Zone Diet® (*P* < .001), and non-coaches were more likely to practice Vegetarian (*P* = .045) diets and Weight Watchers® (*P* = .012). Participants who reported competing, or planning to compete, in competitions were more likely to practice Macro Counting (21.3% vs. 15.4%, *P* < .001) and Renaissance Periodization® (6.2% vs. 3.6%, *P* = .003) than those who were not. Practicing Macro Counting (*P* < .001), Renaissance Periodization® (*P* = .002), and Weight Watchers® (*P* = .041) were also significantly associated with supplement use.

### 3.4 Supplement use and associated factors

A total of 2015 unique supplements were reported. The authors organized each supplement into one of the 26 categories based on the supplement’s ingredients or primary function (Table 3). A description and definition of the supplements that were included in each category may be viewed in Additional File 2. The most frequently reported supplements were protein (*n* = 1320, 51.2%), creatine (*n* = 591, 22.9%), and pre-workout/energy (*n* = 533, 20.7%).

**Table 3.** Supplement use and relationships between sex, coaching status, and practicing a diet.

Supplement	Total % (*n*)	Sex			CrossFit® Coach			Practices a diet		
		Male % (*n*)	Female % (*n*)	*P*	Yes % (*n*)	No % (*n*)	*P*	Yes % (*n*)	No % (*n*)	*P*
Protein	51.2 (1320)	54.1 (670)	48.6 (650)	.005	53.8 (374)	50.3 (946)	ns	53.1 (822)	48.4 (498)	.020
Creatine	22.9 (591)	31.3 (388)	15.2 (203)	<.001	29.8 (207)	20.4 (384)	<.001	25.0 (387)	19.8 (204)	.002
Pre-workout	20.7 (533)	26.7 (331)	15.5 (207)	<.001	23.3 (162)	20.0 (376)	ns	21.4 (331)	20.1 (207)	ns
Multivitamin	17.1 (441)	15.9 (197)	18.2 (244)	ns	17.3 (120)	17.1 (321)	ns	19.2 (297)	14.0 (144)	<.001
Omega FA	15.5 (400)	16.9 (209)	14.3 (191)	ns	22.4 (156)	13.0 (244)	<.001	17.1 (265)	13.1 (135)	.006
Amino Acids	12.8 (329)	12.0 (148)	13.5 (181)	ns	13.4 (93)	12.5 (236)	ns	14.0 (217)	10.9 (112)	.019
Vitamin D	12.4 (319)	10.5 (130)	14.1 (189)	.005	14.5 (101)	11.6 (218)	.044	14.4 (223)	9.3 (96)	<.001
Collagen	11.0 (283)	5.9 (73)	15.7 (210)	<.001	11.5 (80)	10.8 (203)	ns	12.3 (190)	9.0 (93)	.010
Fuel	8.6 (221)	9.0 (111)	8.2 (110)	ns	10.8 (75)	7.8 (146)	.015	9.6 (149)	7.0 (72)	.019
Magnesium	8.0 (206)	5.6 (69)	10.2 (137)	<.001	9.8 (68)	7.3 (138)	.042	9.6 (149)	5.5 (57)	<.001
Joint support	6.6 (171)	6.4 (79)	6.9 (92)	ns	8.2 (57)	6.1 (114)	ns	7.6 (117)	5.2 (54)	.021
Recovery	6.4 (166)	6.7 (83)	6.2 (83)	ns	6.3 (44)	6.5 (122)	ns	6.9 (106)	5.8 (60)	ns
Greens/reds	4.8 (123)	4.1 (51)	5.4 (72)	ns	5.0 (35)	4.7 (88)	ns	5.2 (81)	4.1 (42)	ns
Dig. health	4.4 (114)	2.7 (33)	6.1 (81)	<.001	6.2 (43)	3.8 (71)	.008	5.0 (78)	3.5 (36)	ns
Sleep support	4.1 (106)	3.4 (42)	4.8 (64)	ns	4.3 (30)	4.0 (76)	ns	4.3 (66)	3.9 (40)	ns
B vitamins	3.6 (93)	2.8 (35)	4.3 (58)	.040	4.2 (29)	3.4 (64)	ns	4.5 (69)	2.3 (24)	.005
Herbals	3.5 (90)	2.3 (28)	4.6 (62)	<.001	3.0 (21)	3.7 (69)	ns	4.5 (69)	2.0 (21)	<.001
Zinc	3.2 (82)	3.0 (37)	3.4 (45)	ns	3.9 (27)	2.9 (55)	ns	3.4 (53)	2.8 (29)	ns
Vitamin C	3.0 (77)	2.6 (32)	3.4 (45)	ns	2.6 (18)	3.1 (59)	ns	3.4 (52)	2.4 (25)	ns
Hemp/CBD	2.9 (75)	2.7 (33)	3.1 (42)	ns	4.9 (34)	2.2 (41)	<.001	3.0 (46)	2.8 (29)	ns
Bone support	2.1 (55)	0.9 (11)	3.3 (44)	<.001	1.9 (13)	2.2 (42)	ns	2.3 (35)	1.9 (20)	ns
FB/WM	1.7 (450)	1.4 (17)	2.1 (28)	ns	1.6 (11)	1.8 (34)	ns	1.8 (28)	1.7 (17)	ns
Antioxidant	1.6 (40)	1.2 (15)	1.9 (25)	ns	1.9 (13)	1.4 (27)	ns	1.9 (30)	1.0 (10)	ns
Energy supp.	1.4 (35)	1.6 (20)	1.1 (15)	ns	1.4 (10)	1.3 (25)	ns	1.5 (23)	1.2 (12)	ns
Iron	1.1 (28)	0.4 (5)	1.7 (23)	<.001	0.7 (5)	1.2 (23)	ns	1.4 (21)	0.7 (7)	ns
Test. booster	1.0 (27)	1.7 (21)	0.4 (6)	.002	1.2 (8)	1.0(19)	ns	1.2 (19)	0.8 (8)	ns

Females were more likely than males to consume vitamin D (*P* = .005), collagen (*P* < .001), magnesium (*P* < .001), digestive health supplements (*P* < .001), B vitamins (*P* = .040), herbal mixtures (*P* < .001), bone support mixtures (*P* < .001), and iron (*P* < .001), and males were more likely to consume protein (*P* = .005), creatine (*P* < .001), pre-workout/energy (*P* < .001), and testosterone boosters (*P* = .002). CrossFit® coaches were more likely to consume creatine (*P* < .001), omega fatty acids (*P* < .001), vitamin D (*P* = .044), fuel (*P* = .015), magnesium (*P* = .042), digestive health supplements (*P* = .008), and Hemp/CBD (*P* < .001) than non-coaches. Participants who reported competing, or planning to compete, in competitions were more likely to consume protein (55.8% vs. 45.9%, *P* < .001), creatine (28.8% vs. 16.0%, *P* < .001), pre-workout/energy (26.6% vs. 14.2%, *P* < .001), multivitamin/minerals (18.8% vs. 15.2%, *P* = .017), omega fatty acids (17.8% vs. 12.8%, *P* < .001), amino acids (14.3% vs. 11.0%, *P* = .012), joint support mixtures (8.0% vs. 5.1%, *P* = .003), and recovery supplements (7.5% vs. 5.2%, *P* = .021) than those who did not. Participants who reported practicing a diet were more likely to consume protein (*P* = .020), creatine (*P* = .002), multivitamin/minerals (*P* < .001), omega fatty acids (*P* = .006), amino acids (*P* = .019), vitamin D (*P* < .001), collagen (*P* = .010), fuel (*P* = .019), magnesium (*P* < .001), joint support mixtures (*P* = .021), B vitamins (*P* = .005), and herbal mixtures (*P* < .001) than those who did not.

### 3.5 Associations between dietary practices and supplement use

**Figure 2.**
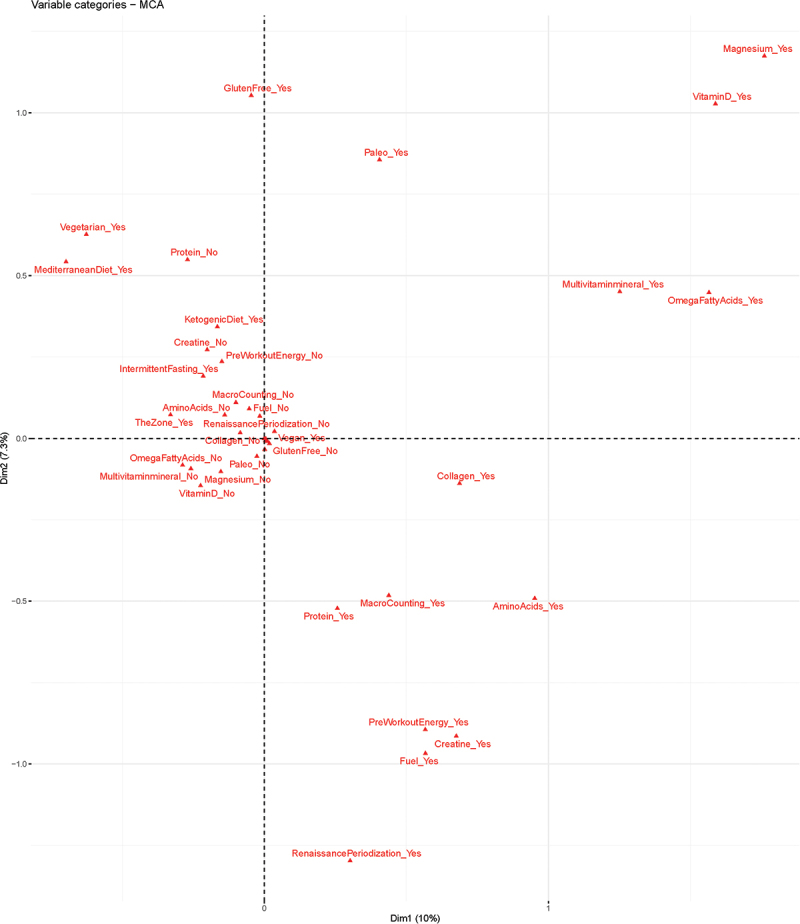
Multiple correspondence analysis.

Graphical representation of MCA between the top 10 diets and supplements is shown in Figure 2. Correspondence revealed three dimensions which explained ~23% of the variation in dietary practices and supplement use. The first dimension was defined as the use of health-related supplements (magnesium, multivitamin/minerals, omega-3 fatty acids, and vitamin D). The second dimension was defined as the use of performance-enhancing or muscle-building supplements (creatine, pre-workout/energy, and protein). The third dimension was defined as ketogenic diet-related factors (ketogenic diet, collagen, and fuel supplements).

The MCA plot showed further general associations with the lower right quadrant appearing to denote diets and supplements associated with physical performance. The upper right quadrant appears to denote diets and supplements related to general health and the upper left quadrant appears to denote diets that emphasize health or fat loss. The categories clustered at the axes’ intersection denote a general eschewing of supplement use.

### 3.6 Dietary and supplement beliefs and associated factors

**Figure 3.**
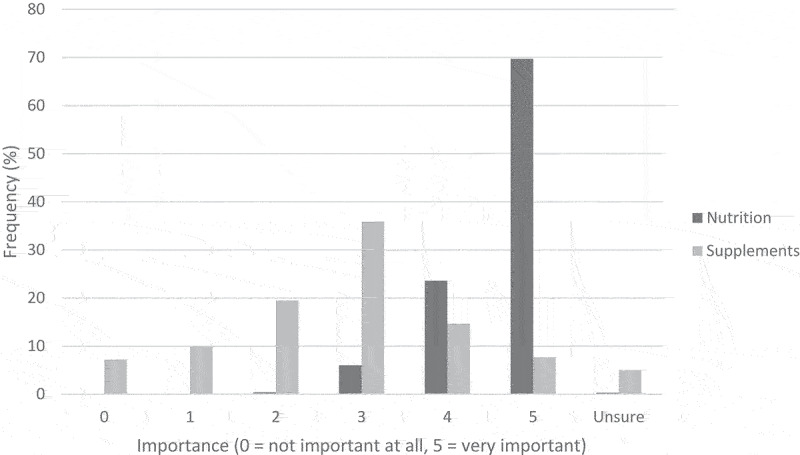
Participant ratings for how important nutrition and supplements are for CrossFit® performance.

Participants’ ratings of the importance of nutrition and supplements for CrossFit® performance are shown in Figure 3. Most participants believed that nutrition was very important for CrossFit® performance, with an average rating of 4.62 on a scale of 0 to 5. Participants tended to believe that supplements were somewhat important for CrossFit® performance, with an average rating of 2.54 on a scale of 0 to 5.

**Figure 4.**
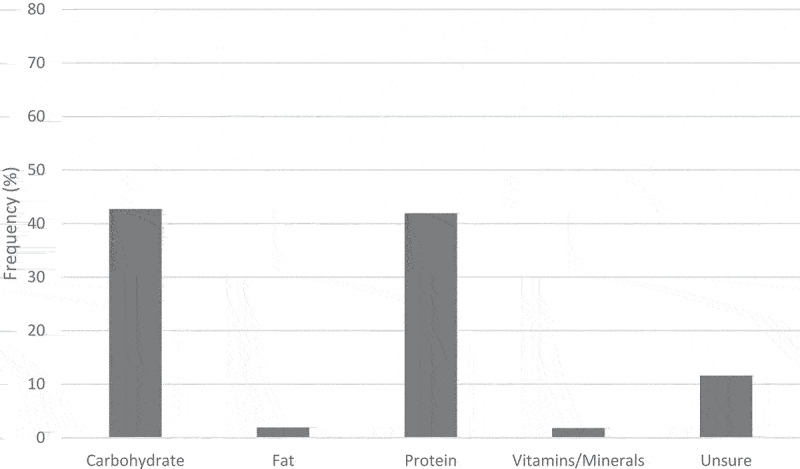
Participant ratings of the most important nutrient for CrossFit® performance.

Participants’ ratings of the most important nutrient for CrossFit® performance are shown in Figure 4. Most participants reported either Carbohydrate (42.7%, *n* = 1099) or Protein (41.9%, *n* = 1079). Females were more likely to select Protein (44.3% vs. 39.3%, *P* < .001) and males were more likely to select Fat (3.2% vs. 0.7%, *P* < .001). CrossFit® coaches were more likely to select Carbohydrate (50.5% vs. 39.8%, *P* < .001) and non-coaches were more likely to select Protein (42.8% vs. 39.6%, *P* < .001). Participants who reported competing or planning to compete were more likely to select Carbohydrates (48.6% vs. 35.7%, *P* < .001) and non-competitors were more likely to select Protein (45.4% vs. 39.0%, *P* < .001). Carbohydrate was more likely to be selected by participants practicing Macro Counting (54.1 vs. 40.1%, *P* < .001), Renaissance Periodization® (58.6% vs. 41.8%, *P* < .001), Vegan (65.8% vs. 42.3%, *P* = .005), or Pescatarian (50.0% vs. 42.6%, *P* = .006) diets. Protein was more likely to be selected by participants practicing Paleo (60.3% vs. 40.7%, *P* < .001) or Ketogenic (57.4% vs. 41.6%, *P* < .001) diets. Fat was more likely to be selected by participants practicing Ketogenic (14.8% vs. 1.7%, *P* < .001) or Pescatarian (9.1% vs. 1.8%, *P* = .006) diets. Vitamins/minerals were also more likely to be selected by participants practicing Ketogenic (7.5% vs. 1.7%, *P* < .001) or Pescatarian (9.1 vs. 1.8%, *P* = .006) diets.

**Figure 5.**
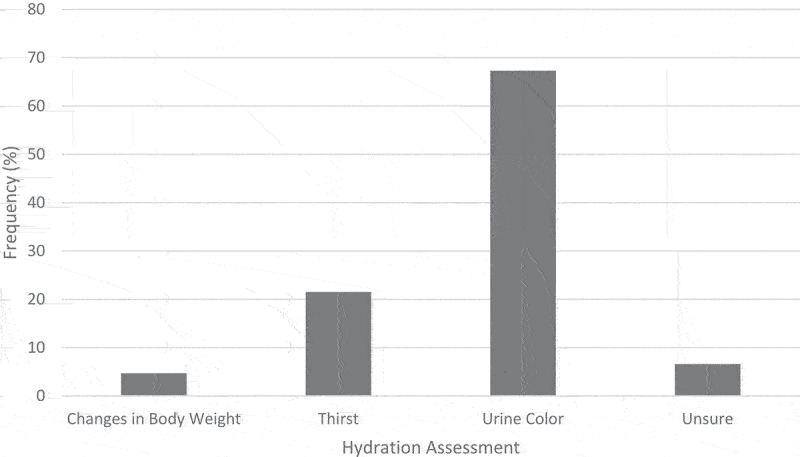
Participant ratings of the most important factor for assessing hydration.

Participants’ ratings of the most important factor for assessing hydration are shown in Figure 5. “Urine Color” was selected by most participants (67.3%, *n* = 1733). Five hundred fifty-four (21.5%) participants selected “Thirst,” 120 (4.7%) selected “Changes in Body Weight,” and 169 (6.6%) selected “Unsure.” Males were more likely to select “Thirst” (25.0% vs. 18.3%, *P* < .001) and females were more likely to select “Urine Color” (71.1% vs. 63.2%, *P* < .001). CrossFit® coaches were more likely to select “Thirst” (28.3% vs. 19.0%, *P* < .001) and non-coaches were more likely to select “Urine Color” (69.4% vs. 61.4%, *P* < .001).

### 3.7 Reasons for practicing a diet and associated factors

**Figure 6.**
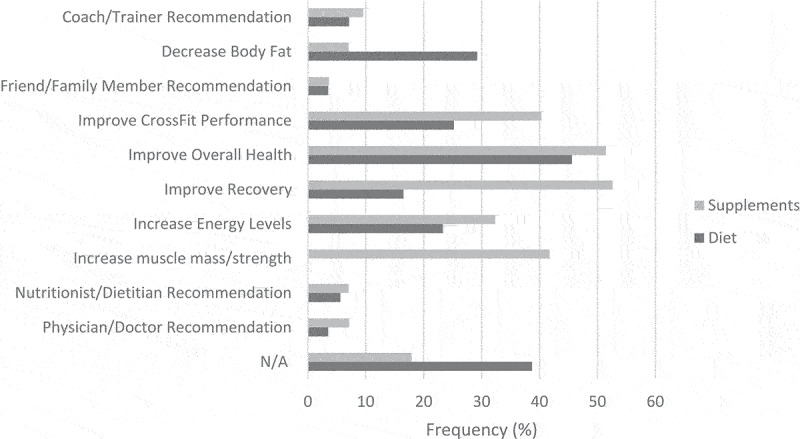
Reasons participants reported for practicing a diet or using supplements.

The reasons participants reported for practicing a diet are shown in Figure 6. The top reasons for practicing a diet were to improve overall health (*n* = 1174, 45.6%), decrease body fat (*n* = 739, 29.2%), and improve CrossFit® performance (*n* = 648, 25.2%). Females were more likely than males to report practicing a diet because of a coach/trainer recommendation (8.5% vs. 5.6%, *P* = .004), to improve overall health (48.0% vs. 43.0%, *P* = .011), to increase energy levels (25.1% vs. 21.2%, *P* = .020), nutritionist/dietitian recommendation (6.8% vs. 4.4%, *P* = .007), or physician/doctor recommendation (4.8% vs. 2.0%, *P* < .001). CrossFit® coaches were more likely to report practicing a diet to improve CrossFit® performance (32.5% vs. 22.4%, *P* < .001), improve overall health (50.2% vs. 43.9%, *P* = .004), improve recovery (20.7% vs. 14.9%, *P* < .001), increase energy levels (27.3% vs. 21.7%, *P* = .003), and less likely report physician/doctor recommendation (2.0% vs. 4.0%, *P* = .015) than non-coaches. Participants who reported competing or planning to compete were more likely to report coach/trainer recommendation (8.0% vs. 6.0%, *P* = .044), improve CrossFit® performance (33.2% vs. 15.7%, *P* < .001), improve recovery (20.8% vs. 11.4%, *P* < .001), increase energy levels (27.2% vs. 18.6%, *P* < .001), and nutritionist/dietitian recommendation (6.6% vs. 4.5%, *P* = .019) than those who did not.

### 3.8 Reasons for using supplements and associated factors

The reasons participants reported for using supplements are shown in Figure 6. The top reasons for using supplements were to improve recovery (*n* = 1355, 52.6%), improve overall health (*n* = 1324, 51.4%), and increase muscle mass/strength (*n* = 1074, 41.7%). Males were more likely to report using supplements to improve CrossFit® performance (50.2% vs. 31.2%, *P* < .001), improve recovery (58.0% vs. 47.7%, *P* < .001), increase energy levels (36.7% vs. 28.2%, *P* < .001), and increase strength/muscle mass (51.1% vs. 33.1%, *P* < .001), and females were more likely to report coach/trainer recommendation (10.8% vs. 8.2%, *P* = .023) and physician/doctor recommendation (9.9% vs. 4.1%, *P* < .001). CrossFit® coaches were more likely to report using supplements to improve CrossFit® performance (47.3% vs. 37.8%, *P* < .001), improve overall health (56.3% vs. 49.7%, *P* = .003), improve recovery (60.6% vs. 49.7%, *P* < .001), increase energy levels (36.4% vs. 30.7%, *P* = .006), and increase strength/muscle mass (45.5% vs. 40.4%, *P* = .020), and non-coaches were more likely to report coach/trainer recommendation (10.4% vs. 7.2%, *P* = .014). Participants who reported competing or planning to compete were more likely to report using supplements to improve CrossFit® performance (52.2% vs. 26.4%, *P* < .001), improve overall health (55.0% vs. 47.3%, *P* < .001), improve recovery (60.2% vs. 43.8%, *P* < .001), increase energy levels (38.4% vs. 25.0%, *P* < .001), and increase strength/muscle mass (49.3% vs. 32.8%, *P* < .001) than those who did not.

### 3.9 Sources of dietary information and associated factors

**Figure 7.**
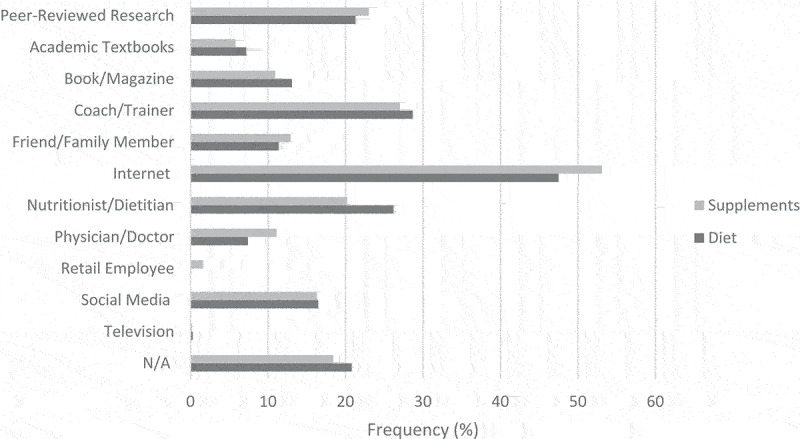
Sources participants reported using to obtain information about diet and supplements.

The sources participants consulted for information on diets are shown in Figure 7. The top sources of dietary information were the Internet (*n* = 1,224, 47.5%), coach/trainer (*n* = 739, 28.7%), and nutritionist/dietitian (*n* = 676, 26.2%). Males were more likely to select academic journals/peer-reviewed research (22.9% vs. 19.7%, *P* = .047), books/magazines (14.6% vs. 11.7%, *P* = .026), and the Internet (50.5% vs. 44.8%, *P* = .004) as sources for dietary information, and females were more likely to select coach/trainer (32.0% vs. 25.1%, *P* < .001) and nutritionist/dietitian (29.4% vs. 22.8%, *P* < .001). CrossFit® coaches were more likely to select academic journals/peer-reviewed research (29.6% vs. 18.2%, *P* < .001), academic textbooks (12.2% vs. 5.3%, *P* < .001), and nutritionist/dietitian (29.4% vs. 25.1%, *P* = .029) and less likely to select physician/doctor (5.6% vs. 8.0%, *P* = .037) than non-coaches. Participants who reported competing or planning to compete were more likely to select academic journals/peer-reviewed research (23.6% vs. 18.5%, *P* < .001), academic textbooks (8.7% vs. 5.4%, *P* < .001), coach/trainer (30.4% vs. 26.7%, *P* = .039), and social media (18.0% vs. 14.6%, *P* = .020) than those who did not.

### 3.10 Sources of information on supplements and associated factors

The sources participants consulted for information on supplements are shown in Figure 7. The top sources of information on supplements were the Internet (*n* = 1368, 53.1%), coach/trainer *(n *= 695, 27.0%), and academic journals/peer-reviewed research (*n* = 592, 23.0%). Males were more likely to select academic journals/peer-reviewed research (27.2% vs. 19.1%, *P* < .001), books/magazines (13.2% vs. 8.8%, *P* < .001), and the Internet (58.3% vs. 48.4%, *P* < .001) as sources of information on supplements, and females were more likely to select physician/doctor (13.8% vs. 8.3%, *P* < .001). CrossFit® coaches were more likely to select academic journals/peer-reviewed research (31.5% vs. 19.9%, *P* < .001), academic textbooks (10.1% vs. 4.3%, *P* < .001), and nutritionist/dietitian (24.2% vs. 18.7%, *P* = .002) as sources of information on supplements, and non-coaches were more likely to select retail employees (0.7% vs. 1.9%, *P* = .037). Participants who reported competing or planning to compete were more likely to select academic journals/peer-reviewed research (26.0% vs. 19.5%, *P* < .001), academic textbooks (7.2% vs. 4.2%, *P* < .001), coach/trainer (30.2% vs. 23.3%, *P* < .001), the Internet (56.2% vs. 49.6%, *P* < .001), nutritionist/dietitian (22.1% vs. 18.0%, *P* = .011), and social media (18.0% vs. 14.3%, *P* = .012) as sources of information on supplements than those who did not.

## 4. Discussion

### 4.1 Dietary practices

One of the primary purposes of this study was to characterize the diets practiced by CrossFit® participants. While there are multiple reports of how popular diets may impact athletic performance [8,25,26], there were previously no data on how many CrossFit® participants practiced these diets. In fact, data on the prevalence of these diets among athletes and the general population are lacking. Most CrossFit® participants in this study (60.1%) reported practicing a specific diet, with Macro Counting (18.6%), Intermittent Fasting (7.7%), and Paleo (6.1%) diets being most prevalent. Interestingly, a relatively small number of participants (1.5%) practiced The Zone Diet®, although it has been recommended by CrossFit® [9]. Maxwell et al. [11] reported that Paleo and The Zone Diet® are commonly recommended by CrossFit® coaches, and CrossFit® coaches in the present study were more likely to practice Paleo and The Zone Diet® than non-coaches. A comprehensive review of the diets reported in this study and their potential effects on health and performance are beyond the scope of this paper. Some recent research on nutrition interventions for CrossFit® is described by de Souza et al. [27], but data on the effects of many of the reported diets on the health and performance of CrossFit® participants are lacking.

### 4.2 Supplement use

Individuals who exercise may use supplements to improve health or performance [16,17], but supplement use among CrossFit® participants was previously unknown. A large proportion of CrossFit® participants (82.2%) reported using at least one supplement twice per week over the past six months. A similar proportion (82.2%) was reported among a sample of youth athletes from various countries [17], college athletes (86%) [18], and high-performance Canadian athletes (87%) [28]. This proportion may be higher than the general population (52–62%) [19,29]. Prevalence of supplement use was higher among males and those who exercised more days per week, which has been reported previously [16,17,30].

The most commonly used supplements were protein (51.2%), creatine (22.9%), and pre-workout/energy (20.7%). The use of protein supplements is highly prevalent among exercising individuals [17,30,31], with one report as high as 80.1% [16]. Creatine use is also prevalent among athletes [17,31]. Males were more likely to consume protein and creatine, which agrees with the findings from Knapik et al. [31]. Most studies on supplement prevalence do not describe the use of pre-workout/energy supplements. However, the prevalence of caffeine and energy drinks may be described, which were included in the present study’s “pre-workout/energy” category (see Additional File 2). Caffeine and energy drink consumption are prevalent among the general population [19,29] and athletes [17].

Relatively few studies have examined the effects of supplements on CrossFit® performance, but interested readers are referred to de Souza et al. [27] for a review of the current scientific literature. To the authors’ knowledge, no studies have been conducted on the effects of creatine for CrossFit® performance. Beta alanine supplementation may also improve performance in high-intensity, short-duration exercise [32], but it has not been extensively studied for CrossFit®. With the high prevalence of supplement use among CrossFit® participants, further research on the effects of supplements on CrossFit® performance is warranted.

### 4.3 Dietary beliefs

Most CrossFit® participants believed that nutrition is very important for CrossFit® performance, which agrees with a previous study on the nutritional beliefs of CrossFit® coaches [11]. There was a relatively even distribution of participants who reported carbohydrate (42.7%) and protein (41.9%) as the most important nutrients for CrossFit® performance. The relative importance of each may largely depend on the training habits and physical goals of each individual athlete. In the present study, participants who reported competing or planning to compete were more likely to select carbohydrate, and participants who were not competing were more likely to select protein.

CrossFit® has advocated for thirst as the optimal measure of hydration [12]. Five-hundred fifty-four (21.5%) participants in the present study reported “Thirst” as the best method for assessing hydration, which was more likely to be reported by CrossFit® coaches than non-coaches. Most participants in this study (67.3%) reported “Urine Color” as the best method to assess hydration, and the smallest proportion of participants (4.7%) selected “Changes in Body Weight.”

### 4.4 Reasons for practicing a diet and sources of information

The top reasons for practicing a diet were to improve overall health (*n* = 1174, 45.6%), decrease body fat (*n* = 739, 29.2%), and improve CrossFit® performance (*n* = 648, 25.2%). Health and fat loss have previously been cited as reasons for dieting among members of the general population [33]. The most common sources of dietary information were the Internet (47.5%), coach/trainer (28.7%), nutritionist/dietitian (26.2%), and academic journals/peer-reviewed research (21.3%). These findings are similar to those of Maxwell et al. [11], who reported that CrossFit® coaches primarily obtained their dietary information from the Internet, peer-reviewed research, and other CrossFit® colleagues.

### 4.5 Reasons for using supplements and sources of information

The top reasons participants reported for using supplements were to improve recovery (52.6%), improve overall health (51.4%), and increase muscle mass/strength (41.7%). These same reasons are often cited in other studies on athletes [18,28] and gym members [16]. Also, in agreement with previous reports, males were more likely than females to select “increase muscle mass/strength” as a reason for using supplements [28,31].

The top sources of information on supplements were the Internet (53.1%), coach/trainer (27.0%), and academic journals/peer-reviewed research (23.0%). The Internet is commonly cited as a source of supplement information for athletes [18,28] and gym members [16]. Coaches and trainers are also cited as sources in similar studies [17,18,28].

### 4.6 Limitations

A limitation of the present study is that the data were self-reported. Data were also collected during the COVID-19 pandemic (May–July 2021). The COVID-19 pandemic may have affected some participants’ workout habits (22.7%), dietary practices (12.9%), or supplement use (6.7%); however, participants were not asked how the pandemic specifically affected these variables. Specific intakes of nutrients and supplement doses were also not reported. However, given the sample size (*n* = 2,576) and diversity (i.e. male/female, age, and geographic reach) of the present study, we believe the findings contribute valuable data to the current body of knowledge on diets and supplements.

## Supplementary Material

Supplemental Material

Supplemental Material
