# Supplementary material for: Dietary practices and supplement use among CrossFit® participants
Source: J Int Soc Sports Nutr. 2022 Jul 4;19(1):316–35. doi: 10.1080/15502783.2022.2086016 (PMC9261745; doi:10.1080/15502783.2022.2086016)
Supplement: Supplemental Material [file RSSN_A_2086016_SM0905.pdf]

If you consent to participate in this survey, please continue to the next page.

1) Please select the state where you primarily perform CrossFit.

If you primarily perform CrossFit outside the United States, please name the country:

2) What is your sex?

- Male
- Female

3) What is your age (in years)?

4) What is your height?

Feet

Inches

5) What is your weight (in pounds)?

6) Are you a CrossFit trainer/coach?

- Yes
- No

7) How long have you been performing CrossFit (in years)?

8) In the past 6 months, on average, how many days per week have you performed CrossFit?

Days per Week

9) In the past 6 months, has the COVID-19 pandemic affected your response to question #8?

- Yes
- No

10) Please select the reason(s) why you perform CrossFit. Select all that apply.

- Health
- Improve/Maintain Physical Appearance
- Fun/Enjoyment
- Social Interaction/Community

11) Do you compete, or are planning to compete, in CrossFit or other fitness competitions?

- Yes
- No

Nutrition Habits

Questions 12-18 will ask you about your current dietary practices.

12) Please select which diet you have **primarily** followed over the past 6 months from the list below. If you have not followed a specific diet, please select 'N/A' at the bottom of the list.

- Atkins Diet
- Carnivore
- DASH Diet
- Dukan

- ○ Flexitarian
- ○ Gluten-Free Diet
- ○ IIFYM (If It Fits Your Macros)
- ○ Intermittent Fasting
- ○ Ketogenic Diet
- ○ Mediterranean Diet
- ○ Paleo
- ○ Pescatarian
- ○ Renaissance Periodization
- ○ South Beach Diet
- ○ The Zone
- ○ Vegan
- ○ Vegetarian
- ○ Weight Watchers
- ○ Whole 9 / Whole 30
- ○ [ ] Other (please specify)
- ○ N/A (I have not followed a specific diet)

13) In the past 6 months, has the COVID-19 pandemic affected your response to question #12?

- ○ Yes
- ○ No

14) If you selected a diet from question #12, what is your primary reason(s) for following that diet? Select all that apply. If you have not followed a specific diet, please select 'N/A' from the bottom of the list.

- ☐ Coach/Trainer recommendation
- ☐ Decrease body fat
- ☐ Friend/Family member recommendation
- ☐ Improve CrossFit performance
- ☐ Improve overall health
- ☐ Improve recovery
- ☐ Increase energy levels

- ☐ Nutritionist/Dietitian recommendation
- ☐ Physician/Doctor recommendation
- ☐ N/A (I don't follow a specific diet)

15) From where do you get most of your information about your diet? Select all that apply.

- ☐ Academic Journals/Peer-Reviewed Research
- ☐ Academic Textbooks
- ☐ Book/Magazine
- ☐ Coach/Trainer
- ☐ Friend/Family member
- ☐ Internet (i.e., Google search, visiting websites)
- ☐ Nutritionist/Dietitian
- ☐ Physician/Doctor
- ☐ Social Media (i.e., Facebook, Instagram, Reddit, YouTube)
- ☐ Television
- ☐ N/A (I'm not seeking information about my diet)

16) On a scale of 0-5, please rate how important you believe nutrition is for CrossFit performance.

- ○ 0 = Not Important at all
- ○ 1
- ○ 2
- ○ 3
- ○ 4
- ○ 5 = Very Important
- ○ Unsure

17) Please select which nutrient you believe is most important for CrossFit performance.

- ○ Carbohydrates
- ○ Fats
- ○ Proteins

- Vitamins/Minerals
- Unsure

18) Please select which factor you believe is most important for assessing hydration.

- Changes in Body Weight
- Thirst
- Urine Color
- Unsure

Supplement Habits

Questions 19-23 will ask you about your use of dietary and sports supplements over the past 6 months. Dietary and sports supplements are products consumed in the form of tablets, capsules, softgels, gelcaps, liquids, powders, or gummies with the purpose of improving health and/or performance.

Examples of dietary and sports supplements include vitamins, minerals, amino acids, creatine, protein powders, fish/omega-3 oils, fat burners, pre-workouts, probiotics, mixtures of natural ingredients to support health (adrenal, brain, immune system, joint, or sleep support), or other natural substances (CBD oil, turmeric, melatonin, glucosamine, beetroot juice, tart cherry, green tea extract, etc.).

19) Please write the **name** and **brand** of all dietary and sports supplements you have consumed **at least two days per week** over the past 6 months.

20) In the past 6 months, has the COVID-19 pandemic affected your response to Question #19?

- Yes
- No

21) If you consume supplements, what is your primary reason(s)? Select all that apply. If you have not consumed supplements, please select 'N/A' from the bottom of the list.

- ☐ Coach/Trainer recommendation
- ☐ Decrease body fat
- ☐ Friend/Family member recommendation
- ☐ Improve CrossFit performance
- ☐ Improve overall health
- ☐ Improve recovery
- ☐ Increase energy levels
- ☐ Increase muscle mass/strength
- ☐ Nutritionist/Dietitian recommendation
- ☐ Physician/Doctor recommendation
- ☐ N/A (I don't currently use supplements)

22) From where do you get most of your information about supplements? Select all that apply.

- ☐ Academic Journals/Peer-Reviewed Research
- ☐ Academic Textbooks
- ☐ Book/Magazine
- ☐ Coach/Trainer
- ☐ Friend/Family Member
- ☐ Internet (i.e., Google search, visiting websites)
- ☐ Nutritionist/Dietitian
- ☐ Physician/Doctor
- ☐ Retail employee
- ☐ Social Media (i.e., Facebook, Instagram, Reddit, YouTube)
- ☐ Television
- ☐ N/A (I am not seeking information about supplements)

23) On a scale of 0-5, please rate how important you believe supplements are for CrossFit performance.

- ○ 0 = Not important at all
- ○ 1
- ○ 2
- ○ 3

- 4
- 5 = Very Important
- Unsure

Proceed to the next page to submit the survey.

Powered by Qualtrics
