# Supplementary material for: Dietary practices and supplement use among CrossFit® participants
Source: J Int Soc Sports Nutr. 2022 Jul 4;19(1):316–35. doi: 10.1080/15502783.2022.2086016 (PMC9261745; doi:10.1080/15502783.2022.2086016)
Supplement: Supplemental Material [file RSSN_A_2086016_SM0902.docx]

Additional Table 1. Use of nutrition replacement supplements reported by CrossFit participants.

| Supplement | Frequency % (n) |
| --- | --- |
| Protein | 51.2% (1320) |
| Protein – Bar | 0.8% (21) |
| Protein – Beef | 0.2% (4) |
| Protein – Blend^1^ | 2.0% (51) |
| Protein – Bone Broth | 0.2% (6) |
| Protein – Casein | 4.0% (102) |
| Protein – Egg White | 0.3% (7) |
| Protein – Plant-based | 5.1% (131) |
| Protein – Whey | 30.9% (797) |
| Protein – Not Specified^2^ | 13.4% (344) |
| Multivitamin/mineral | 17.1% (441) |
| Biotin | 0.7% (18) |
| Folate | 0.3% (7) |
| Hair/Skin/Nail Support^3^ | 0.3% (9) |
| Multivitamin | 14.2% (365) |
| Mineral mixtures | 0.8% (20) |
| Organ^4^ | 0.5% (14) |
| Vitamin Packs^5^ | 0.5% (14) |
| ZMA^6^ | 1.0% (27) |
| Omega Fatty Acids | 15.5% (400) |
| Fish Oil | 14.1% (362) |
| Krill Oil | 0.8% (20) |
| Omega Oil Blend | 0.6% (15) |
| Vegan Omega | 0.2% (6) |

^1^Mixtures of different protein sources; ^2^Some brands sell protein from different sources, and this category was used when participants did not specify which one; ^3^Mixtures of vitamins and minerals marketed to support hair, skin, or nail health; ^4^Example: powdered beef liver; ^5^Multivitamins with added herbals and/or other animal products; ^6^Zinc-Magnesium-Vitamin B6

Additional Table 1. Use of nutrition replacement supplements reported by CrossFit participants. (*continued*)

| Supplement | Frequency % (n) |
| --- | --- |
| Amino Acids | 12.8% (329) |
| 5-HTP | 0.2% (4) |
| Aspartic Acid | 0.1% (1) |
| Branched Chain Amino Acids | 9.5% (245) |
| Carnitine | 0.5% (14) |
| Essential Amino Acids | 1.8% (46) |
| GABA | 0.2% (5) |
| Glutamine | 1.0% (27) |
| Glycine | 0.1% (1) |
| Lysine | 0.2% (6) |
| HMB | 0.3% (8) |
| Leucine | 0.1% (2) |
| Lysine | 0.2% (6) |
| NAC | 0.3% (7) |
| Theanine | 0.1% (1) |
| Vitamin D | 12.4% (319) |
| Vitamin D | 11.6% (298) |
| Vitamin D+K | 0.8% (21) |
| Magnesium | 8.0% (206) |
| Greens/Reds^7^ | 4.8% (123) |
| Greens | 4.6% (119) |
| Reds | 1.4% (37) |
| B Vitamins | 3.6% (93) |
| Vitamin B Complex | 1.8% (46) |
| Vitamin B12 | 1.9% (49) |
| Zinc | 3.2% (82) |
| Vitamin C | 3.0% (77) |
| Iron | 1.1% (28) |

^7^Powdered blends of vegetables and/or fruits that may include digestive enzymes or probiotics

Additional Table 2. Use of general health supplements reported by CrossFit participants.

| Supplement | Frequency % (n) |
| --- | --- |
| Collagen | 11.0% (283) |
| Joint Support | 6.6% (171) |
| Glucosamine mixtures^1^ | 2.6% (68) |
| Joint Support mixtures^2^ | 1.4% (36) |
| Turmeric | 3.4% (87) |
| Digestive Health | 4.4% (114) |
| Digestive Support mixtures^3^ | 1.2% (30) |
| Probiotics/Prebiotics/Synbiotics | 3.6% (92) |
| Sleep Support | 4.1% (106) |
| Melatonin | 2.2% (57) |
| Sleep Support mixtures^4^ | 1.9% (50) |
| Herbals | 3.5% (90) |
| Adrenal Support mixtures^5^ | 0.3% (8) |
| Ashwagandha | 1.3% (34) |
| Cholesterol Support mixtures^5^ | 0.3% (8) |
| Cognitive Support mixtures^5^ | 0.5% (12) |
| Detox/Cleanse mixtures^5^ | 0.1% (2) |
| Maca | 0.2% (5) |
| Milk Thistle | 0.2% (5) |
| Mushroom blends^6^ | 0.3% (8) |
| Stress Support mixtures^5^ | 0.3% (7) |
| Thyroid Support mixtures^5^ | 0.3% (7) |
| Women’s Health mixtures^5^ | 0.5% (14) |
| Hemp/CBD | 2.9% (75) |
| Bone Support | 2.1% (55) |
| Bone Support mixtures^7^ | 0.5% (13) |
| Calcium | 1.6% (42) |
| Fat Burner/Weight Management | 1.7% (450) |
| Apple Cider Vinegar | 0.9% (23) |
| Fat Burner/Metabolic Booster mixtures^8^ | 0.7% (18) |
| Weight Management mixtures^9^ | 0.3% (9) |

^1^Includes chondroitin or MSM; ^2^Mixtures including glucosamine, turmeric, and/or other herbals marketed to support joint health; ^3^Digestive enzymes and/or fiber; ^4^Mixtures of melatonin and other herbals or CBD; ^5^Herbal mixtures were placed into categories based on their marketed purpose; ^6^Mixtures of powdered mushrooms, such as Cordyceps and Shiitake; ^7^Mixtures of calcium, magnesium, and/or vitamin D; ^8^Mixtures of caffeine, vitamins and/or herbals marketed for energy or fat loss; ^9^Mixtures of CLA, caffeine, herbals, and/or green tea marketed for weight control

Additional Table 2. Use of general health supplements reported by CrossFit participants. (*continued*)

| Supplement | Frequency % (n) |
| --- | --- |
| Antioxidant | 1.6% (40) |
| Alpha Lipoic Acid | 0.2% (5) |
| Antioxidant mixtures^10^ | 0.8% (20) |
| Glutathione | 0.1% (3) |
| Selenium | 0.1% (3) |
| Vitamin E | 0.4% (10) |
| Energy Support | 1.4% (35) |
| CoQ10 | 1.2% (30) |
| Energy Support mixtures^11^ | 0.2% (6) |

^10^Mixtures including resveratrol, tart cherry, DIM, and others marketed as antioxidants; ^11^Mixtures including vitamins, NADH, and/or ribose marketed to support energy levels

Additional Table 3. Use of performance-enhancing supplements reported by CrossFit participants.

| Supplement | Frequency % (n) |
| --- | --- |
| Creatine | 22.9% (591) |
| Pre-Workout/Energy | 20.7% (533) |
| Pre-Workout (with caffeine)^1^ | 11.1% (286) |
| Beta Alanine | 5.8% (150) |
| Energy Drink^2^ | 2.2% (57) |
| Pre-Workout (without caffeine) | 1.9% (49) |
| Pre-Workout (not specified)^3^ | 1.2% (32) |
| Beetroot Extract | 1.0% (25) |
| Caffeine | 0.5% (14) |
| Arginine | 0.3% (8) |
| Citrulline | 0.2% (4) |
| Fuel | 8.6% (221) |
| Electrolytes | 3.5% (91) |
| Carbohydrates | 2.1% (54) |
| Electrolyte + Carbohydrate mixtures | 1.2% (30) |
| Medium-Chain Triacylglycerols | 0.9% (24) |
| Intraworkout mixtures^4^ | 0.8% (20) |
| Meal Replacement^5^ | 0.8% (21) |
| Recovery^6^ | 6.4% (166) |
| Testosterone Booster^7^ | 1.0% (27) |

^1^Pre-Workouts are powdered mixtures of amino acids, herbals, and/or other food products marketed for consumption before exercise; ^2^Liquids containing caffeine, B Vitamins, and/or other ingredients marketed to increase energy levels; ^3^Some brands sell Pre-Workout powders with or without caffeine, and supplements were categorized here when the participant did not specify which one; ^4^Mixtures of amino acids, carbohydrates, and/or electrolytes that are marketed for consumption during exercise; ^5^Mixtures of carbohydrates, proteins, and fats in liquid or powder form; ^6^Mixtures of amino acids, carbohydrates, protein, and/or electrolytes that are marketed for consumption post-exercise; ^7^Examples: Deer Antler Velvet, DHEA, Fenugreek, Tribulus, and Ecdysterone
